# COVID-19 Health Passes: Practical and Ethical Issues

**DOI:** 10.1007/s11673-022-10227-2

**Published:** 2023-01-11

**Authors:** Gustavo Ortiz-Millán

**Affiliations:** Instituto de Investigaciones Filosóficas, Universidad Nacional Autónoma de México (UNAM), Circuito Mario de la Cueva s/n, Ciudad Universitaria, Coyoacán, 04510 Mexico City, Mexico

**Keywords:** COVID-19, COVID passes, Health certificates, Vaccination, Freedom, Discrimination

## Abstract

Several countries have implemented COVID-19 health passes or certificates to promote a safer return to in-person social activities. These passes have been proposed as a way to prove that someone has been vaccinated, has recovered from the disease, or has negative results on a diagnostic test. However, many people have questioned their ethical justification. This article presents some practical and ethical problems to consider in the event of wishing to implement these passes. Among the former, it is questioned how accurate diagnostic tests are as a means of ensuring that a person is not contagious, whether vaccination guarantees immunity, the fact that health passes can be forged, whether they encourage vaccination, and the problem that there is no universally recognized health pass. Among the ethical issues, it is discussed whether health passes promote discrimination and inequality and whether they violate rights to privacy and freedom. It is concluded that health passes have enough ethical justification to be implemented.

## COVID-19 Health Passes: A Ticket Back to Normal?

With the intention of promoting a safe return to in-person social activities, as well as economic recovery after the crisis brought on by the COVID-19 pandemic, many countries have decided to implement health passes or certificates, through which people could prove that they have been vaccinated, that they have recovered from the disease, or that they have negative results in a diagnostic test obtained recently. This would allow us to safely return to offices, shops, restaurants, gyms, sporting events, shows, universities, and other places where there is social contact and a greater risk of catching and spreading the disease. From February to September 2021, 129 countries request some type of vaccination certificate from their population or from those who want to enter their territory without having to isolate themselves or comply with any quarantine (Howell 2021).

However, many people around the world have wondered if such health passes are ethically justified. The British Parliament received a petition, signed by more than 375,000 people, urging them to reject any type of vaccination passport. “We want the Government to commit to not rolling out any e-vaccination status/immunity passport to the British public. Such passports could be used to restrict the rights of people who have refused a COVID-19 vaccine, which would be unacceptable” (Petitions 2021 ¶1). In France, after weeks of local protests by several tens of thousands of protesters, on August 27 some 160,000 people demonstrated in different cities to protest against the imposition of the *passe sanitaire*, promoted by President Macron, arguing that it unfairly restricted those who have not been vaccinated (AFP 2021). In Italy, thousands have demonstrated against the “green pass,” which the government has imposed as a mandatory condition on all workers, public and private, as of October 15 (Roberts and Martuscelli 2021). All of these people oppose COVID-19 vaccination certificates because they see them as an unjustified restriction of freedom, as a violation of the right to privacy, and as a discriminatory measure. Thus, it is worth asking: Is the imposition of these health passes ethically justified? I shall answer this question by looking at both the practical and the ethical considerations surrounding the implementation of such passes.

Before evaluating the ethics of COVID-19 health passes, we have to know what they are and how they work. First of all, it should be said that health certificates of this type are not new. Quarantines have been established since ancient times to limit the spread of infectious diseases; therefore, documents have been necessary to certify that a person has completed the quarantine, has been vaccinated, or is not contagious. The first records of health certificates date back to the times of the Black Death in the fourteenth century, when they were needed particularly in the context of trade within Europe. The intention was that merchants and other people who had to travel to other cities should not spread the disease and introduce it to cities where there was none (Bamji 2019). Upon arriving in Verona in 1580, Michel de Montaigne reports that “without the *bollette della Sanità*, which they had obtained in Trento and validated in Rovereto, they would not have entered the city, although there was no rumour of a danger of plague” (Montaigne 1889, 118). In *A Journal of the Plague Year*, Daniel Defoe also tells us that during the 1665 plague in London “there were such pressing and crowding there to get passes and certificates of health for such as travelled abroad, for without these there was no being admitted to pass through the towns upon the road, or to lodge in any inn” (Defoe 1722, “Plague, 2. Parishes infected, 1,” ¶18).

More recently, other types of vaccination certificates have been implemented, such as the International Certificate of Vaccination and Prophylaxis, also called the “yellow card,” instituted by the World Health Organization (WHO) in the 1930s with the purpose of serving as proof of vaccination against yellow fever and preventing this disease from leaving countries where it is endemic through foreign travel. The public health purposes of this certificate are different from the case of COVID-19, a disease that is spread from person to person globally. There are other significant differences with the yellow card: there is only one vaccine for yellow fever and it has an efficacy rate of 99 per cent, in addition to having permanent validity, since the vaccine for yellow fever generates immunity for life, while for COVID-19 there are several vaccines with different levels of efficacy and with a limited duration of immunity (Kofler and Baylis 2021). Unlike the proposed COVID-19 health passes, all WHO member countries recognize the yellow card. However, in the absence of a digital COVID-19 health pass, the WHO recommends that vaccination status be recorded in the yellow card (WHO 2021a, xiii).

COVID-19 health passes (also known as vaccination, COVID-19, or health certificates, or as Coronapass, green pass, or COVID passports) are digital apps or printed documents through which governments certify that the bearer has been vaccinated against the SARS-CoV-2 virus, has received negative results in a diagnostic test performed one to three days before, or has recovered from the disease and has a high level of immunity (this is also different from the yellow card, which is only a vaccination, not an immunity, certificate). These certificates are usually issued by government agencies or ministries of health, or by state governments, such as the New York state government, which has issued the so-called Excelsior pass (NYS 2021). The European Commission has also created a digital certificate, called the Digital Green Certificate, for citizens and residents of the countries of the European Union (EC 2021). The first country to implement a COVID-19 vaccination certificate for its citizens was Israel, in February 2021.

These passes should meet the specific public health needs of each country, and states should be clear about what uses are proposed and that these passes should not be used for other purposes. In general, countries’ public health goals and justification are that passes be used as proof of vaccination or immunity; these countries’ ultimate goal is to reduce severe disease cases, prevent health systems from becoming overwhelmed, and return to social life in a safe environment. They may also require them for travellers so that authorities can control the entry of people who may represent a risk to the public health of the country. But the uses that countries have assigned to health passes differ. Some, like Estonia, only ask for them to enter the country; others, such as Israel or Denmark, ask for them in order to access public places, such as restaurants, cinemas, and nightclubs (Murphy 2021). Italy has made them mandatory for workers in the public and private sectors.

Some certificates have been created by private companies and non-profit associations, as well as by associations whose interest is not public health but promoting the safety and trust of their customers, such as the International Air Transport Association (IATA 2021), which promotes their use among its 290 partner airlines in order to provide safety for travellers. All these certificates are digital and can be obtained through a mobile phone app.

The WHO first stated that it did not support the idea that national authorities require vaccination passports for international travel, since there was no guarantee that they would prevent the spread of the virus but also because they could generate inequalities among people given the unequal distribution of vaccines globally (WHO 2021b). However, the WHO itself created a Working Group for a Smart Vaccination Certificate (in which UNICEF, the European Commission, and the International Telecommunications Union participate) to establish standards and specifications for a digital certificate of vaccination that could be internationally recognized (WHO 2021c). This group was dissolved in June 2021. Then in August 2021 the WHO published its guidance on “Digital documentation of COVID-19 certificates: vaccination status” (WHO 2021c), which includes the ethical and technical considerations that countries should take into account if they want to implement these certificates.

I shall analyse some of the practical problems faced by the implementation of COVID-19 health passes as well as some of the ethical problems raised by this implementation in order to later analyse the ethical problems they present.

## Practical Considerations

### Accuracy of Diagnostic Tests

Many of the COVID-19 health passes that have been implemented, such as that of the European Union, record the results of diagnostic tests, so it must be taken into account that these tests have margins of error and depend on many factors, such as the type of test, the development time of the disease, and the quality of the sample, as well as the biology of the patient. They also depend on the sensitivity of the test, that is, its ability to detect a positive case, and its specificity, its ability to determine a negative case. A high-sensitivity test is less likely to provide a false negative, and a high-specificity test is less likely to provide a false-positive result.

There are basically three types of diagnostic tests. The first is nucleic acid amplification tests (NAATs) that detect the genetic material of the SARS-CoV-2 virus by molecular methods such as polymerase chain reaction (PCR). This test is very specific because it is based on the unique genetic sequence of SARS-CoV-2. If a test is positive, there is a high probability that SARS-CoV-2 viral RNA is present in the sample obtained. However, if the sample is not taken from the nasopharynx, where there is a high concentration of viral load, then it is likely to result in a false negative, that is, a test saying that someone does not have the virus when she actually does. Such a person could easily spread the virus because she acts under the belief that she does not have it. The false negative rate also varies according to the length of time the infection has been present, as the viral load varies during the course of the disease. In one study (Kucirka et al. 2020), the false negative rate was 20 per cent when the test was performed five days after symptoms began. The rate was much higher when done earlier in the development of the disease (up to 100 per cent). Another problem is that this type of test can continue to give positive results long after the person is no longer contagious and is not at risk of infecting others (CDC 2021a).

The second type is the antigen test, which seeks to identify one of the outer proteins of the viral coat or envelope, and is more likely to have a false negative result. How reliable are rapid tests? In a study published in March 2021 by the Cochrane COVID-19 Diagnostic Test Accuracy Group, antigen tests—which can give results fifteen minutes after the sample is taken—correctly identified the infection in people with confirmed COVID-19 in an average of 72 per cent of people with symptoms, compared with 58 per cent of people without symptoms. The tests were most accurate when performed during the first week after the first symptoms because the viral load is highest during that period. In people who were confirmed not to have COVID-19, antigen tests correctly ruled out infection in 99.5 per cent of people with symptoms and 98.9 per cent of people without symptoms (Dinnes et al. 2021). Thus, the probability of error is low in the cases in which there is no infection, while it is high in the cases of infected people (less when there are symptoms than when there are none), so the results of these tests are to be distrusted. The sensitivity of rapid tests may also vary with SARS-CoV-2 variants, such as the Omicron variant. A study found that rapid tests were not as sensitive in picking up Omicron infections when viral load was lower, missing about 35 per cent of people who had a positive PCR with any level of virus (Schrom et al. 2022).

The third type of test detects whether the body has developed antibodies. This test shows if the individual has developed an immune response to the SARS-CoV-2 virus; however, antibodies to COVID-19 may not be identifiable in the blood until days or weeks after the person has been infected (Petherick 2020). On the other hand, it is difficult to do a serological test for COVID-19 that has high specificity and sensitivity and that identifies SARS-CoV-2 and not other coronaviruses present, for example, in other types of common colds, which could result in many false positives, that is, the test may report that a person has the virus when in fact he does not. The result of the test can vary over time: if it is done too early, the probability of false negatives is high. The false negative rate is 20 per cent. However, the false negative range is zero to 30 per cent, depending on the brand of the test, as well as the moment in the course of infection when the test is performed. There is research suggesting that antibody levels can decline rapidly, within a few months (Ibarrondo et al. 2020). A positive antibody test shows that the person has been exposed to the SARS-CoV-2 virus; however, it is not certain whether this result indicates a lack of contagion or a long-lasting protective immunity (Schmerling 2021).

COVID-19 health passes should specify the type of test that the person had, as well as the estimated period of validity of the test. In general, it is suggested that in case of travel, the tests have been carried out one to three days before.

To be ethical, health passes must be based on a principle of scientific validity—an evaluation of whether these passes are effective and whether the information they are based on is accurate. As shown, diagnostic tests have high levels of accuracy, but given that they have considerable margins of error, it would be convenient not to depend on them for the issuance of health passes, or to request that they be supported by proof of vaccination.

### Vaccination Is not Synonymous with Immunity

Vaccines are a means of achieving immunity, but it is not correct to call them “immunizations.” Immunity can be achieved by other means, such as having contracted a disease and having recovered and created antibodies, which make us immune. However, this does not happen with all diseases and particularly not with COVID-19. Getting sick and recovering from illness does not guarantee immunity. Having been vaccinated also does not guarantee that one is immune to the disease; none of the currently approved vaccines is a hundred per cent effective in preventing the disease. In both cases, the person has acquired a degree of immunity but is not completely exempt from contracting the disease or from being a carrier of the virus and from spreading it.

Those who argue against COVID-19 health passes claim that a person can have a pass that shows that they have been fully vaccinated and still be a carrier of the virus and infect others or become ill. However, as of October 12, 2021, more than 187 million people had been fully vaccinated in the United States, of whom 31,895 were infected with the virus (what is known as vaccine breakthrough infections) and were hospitalized or had died, according to the Centers for Disease Control and Prevention (CDC 2021b). Many of them belonged to some high-risk group, such as people with comorbidities or seniors. This represents 0.017 per cent of all vaccinated people. In other words, although it does not achieve one hundred per cent, vaccination prevents most people from getting sick and dying; and if they do get sick, there is a high probably that the sickness will not be serious. On the other hand, an estimated 98.6 per cent of people hospitalized with a COVID-19 diagnosis between June and August 2021 were not vaccinated (Amin and Cox 2021).

Thus, a COVID-19 health pass that demonstrates that the person complied with her full vaccination schedule would guarantee that at least that person has a low risk of infection, hospitalization, and death. With a lower risk of infection, the risk of infecting others also decreases, and then the purpose of the health pass is fulfilled so that there can be social interaction again without (or with low) risk of contagion.

Given that current COVID-19 vaccines provide variable and incomplete protection against infection and transmission but are highly effective at preventing severe illness, the primary public health aim of COVID-19 vaccination passes in some places is to reduce severe cases so as to reduce the strain on health systems rather than to prevent transmission. Thus, the justification of COVID-19 vaccination certificates may not be the same as for certificates based on testing.

### Immunity After Infection, Vaccines, and Variants

The development of approved vaccines has been so rapid that we do not know with certainty how long immunity lasts after vaccination with each of the available vaccines, so immunity could be lost before the COVID-19 health pass expires and people who have it may not be as protected as they think and so could spread the virus. There are also doubts about whether approved vaccines are effective in protecting us from virus variants, such that one could have a health pass and not be protected against variants.

Regarding the levels of immunity after the disease and the probability of being reinfected, several studies have shown a very low probability of reinfection, which reveals high rates of immunity. A study conducted with more than 9,000 recovered COVID-19 patients through November 2020 showed a reinfection rate of only 0.7 per cent (Qureshi et al. 2021). Another more recent study states that there is a 95 per cent protection rate in patients who have recovered and that this protection lasts up to seven months (Abu-Raddad et al. 2021a, b; Hall et al. 2022). In addition, if a person who has recovered is vaccinated, she gains “hybrid immunity,” in which twenty-five to one hundred more antibody responses are obtained (Crotty 2021).

Immunity levels after vaccination vary depending on the type of vaccine, but they are generally high (between 65 and 95 per cent efficacious). Some studies suggest that the effectiveness of mRNA vaccines, such as Pfizer/BioNTech, have decreased after seven months (UKHSA 2022; Levin et al. 2021; Hall et al. 2022). Viral vector vaccines (such as AstraZeneca and Johnson & Johnson) are effective at preventing infection and hospitalizations for at least six months (Bartlett 2021; Polinski et al. 2022).

Regarding variants of the virus, such as Alpha, Delta, and Omicron, these are more transmissible, and some are potentially more dangerous. One study indicates that vaccines approved for the parent virus have shown slight differences in efficacy against Delta and Alpha variants after receiving the full vaccination schedule (Lopez Bernal et al. 2021). However, another study published in July 2021 in the *New England Journal of Medicine* suggests that the efficacy of the Pfizer-BioNTech vaccine in preventing infection in vaccinated people was reduced from 89.5 to 75 per cent when faced with the Beta variant (B.1.351) (Abu-Raddad, Chemaitelly, and Butt 2021). As for the Omicron variant, the Pfizer-BioNTech vaccine has an effectiveness of 70 per cent in preventing hospitalizations for COVID-19 (Collie et al. 2021).

COVID-19 health passes would have to consider the most up-to-date information about the periods of natural immunity after recovery from the disease, as well as those of each vaccine, so that passes must have time limits and will have to be renewed with each booster shot. Periodic booster shots are likely to be necessary, as is the case with the flu vaccine.

### Health Passes Can Be Forged

There are data that have been recorded for more than six centuries on falsifications of health certificates (Bamji 2019). This has forced health authorities to employ more sophisticated methods to prevent these frauds. When the CDC implemented an official vaccination card for COVID-19, counterfeits began to be sold online. Members of anti-vaccine groups acquired them and claimed to have them through their websites or social networks. A New Jersey woman sold several hundred fake COVID-19 vaccination cards at US$200 each to people in the New York City area, including hospital and nursing home staff (AP 2021). Similar cases have been detected in other U.S. states, as well as other countries, and although counterfeiters and their buyers have been prosecuted, by the time this happens there are already many fake vaccination cards on the street. Governments should take measures, such as implementing cards with anti-forgery ink and paper, so that they are not so easily forged.

Something similar happens with digital COVID-19 health passes and their apps. Both in Canada and Australia, online sites have been detected that offer both false vaccination cards and QR codes generated by apps similar to those of official issuing agencies (Thompson 2021; Creedon 2021). If COVID-19 health passes are to be implemented, they should be designed with high levels of encryption, which is something that several developers are working on (Greig 2021). However, if tech companies like Yahoo or big banks have been hacked in the past, it is not unlikely that national health systems are hacked as well and that fake COVID-19 health passes can be created and even registered in the national databases of such passports. However, it is unlikely that many people risk getting a fake health pass in the first place because, in some countries like Australia or the United States, people caught selling or using these fake certificates risk high fines and/or sentences ranging from one to ten years in prison (Robertson and Oliver 2021). In addition, people who acquired them would have to be getting them every time a renewal is required. If in fact they are a minority—who would not be protected—then it is likely that health passes guarantee protection to the majority who would have obtained their health passes legally.

### Do Health Passes Encourage Vaccination?

Many people fear that if COVID-19 health passes are implemented or become mandatory, this may be counterproductive and exacerbate the antagonism and resistance that have emerged in many societies around the issue of vaccination and give rise to more mistrust among individuals who are already concerned about the mechanisms of public coercion and the supposed violation of their rights and freedoms. Some have argued that these passes would not encourage vaccination, and opinion surveys seem to support this claim. For instance, in the iCare study at the Montreal Behavioral Medicine Center, conducted between March and June 2021, 30 per cent of the participants stated that introducing vaccination certificates would make it more likely that they would get vaccinated; however, 63 per cent said it would have no influence on their decision, and 4 per cent said it would make it less likely that they would get vaccinated (MBMC 2021).

However, these fears have been proven unjustified by a study that shows that the introduction of health passes does encourage vaccination. In the most comprehensive empirical study so far, Mills and Rüttenauer compared six countries (Denmark, Israel, Italy, France, Germany, and Switzerland) that introduced certification between April and August 2021 with nineteen control countries. They found that

COVID-19 certification led to increased vaccinations 20 days before implementation in anticipation, with a lasting effect up to 40 days after. Countries with pre-intervention uptake that was below average had a more pronounced increase in daily vaccinations compared with those where uptake was already average or higher. (Mills and Rüttenauer 2022)

These findings have clear ethical implications, since increasing vaccination protects more people—not only those who are vaccinated but also those who are not—by reducing transmission and risk of serious illness and death.

### The Health Passes Are not Universal: They Are not Recognized by all Countries

As of April 2022, there are nineteen vaccines authorized for emergency use by different countries, only eight of which have been authorized by the WHO (COVID-19 Vaccine Tracker 2022); twelve have been fully approved by at least one country (Zimmer et al., 2022). The problem for COVID-19 health passes lies in one of the forms that the so-called “vaccine nationalism” has taken, which is that there is not universal recognition of all vaccines and that many countries selectively recognize the vaccines produced in the country and not those of other countries. Thus, the United States recognizes vaccines authorized by the Food and Drug Administration (FDA) and by the WHO (Moderna, Pfizer, Janssen, AstraZeneca, Covishield, Sinopharm, Sinovac, and Covaxin) but not many Chinese vaccines (such as CanSino or ARCoV, among others) or Russia’s Sputnik V. The The United States has imposed travel restrictions on people vaccinated with these vaccines (CDC 2022a). Thus, COVID-19 health passes are not suitable for international travel, except in contexts such as the European Union, which recognizes a single digital certificate for citizens and residents of its twenty-seven member countries (EC 2021). However, the world is far from having a universal standard that serves as a test for crossing borders, and as long as there is no such standard, COVID-19 health passes will be very limited outside the national context. For these reasons, the eighth meeting of the Murphy (2021) Emergency Committee on COVID-19, held on July 14, 2021, advised that in the context of international travel, countries should not require proof of COVID-19 vaccination as a condition for travel (WHO 2021d). Thus, one of the purposes of COVID-19 health passes is not fulfilled, which is to allow vaccinated people to travel internationally.

## Ethical Considerations

There are several ethical issues involved in the implementation of COVID-19 health passes. As is the case with other public health measures implemented during the pandemic, these passes should follow ethical principles of necessity, proportionality, scientific validity, and time boundedness (Morley et al. 2020). The principle of necessity states that governments should only interfere with a person’s rights when considered essential for public health interests. As already argued, health passes help to reduce severe disease cases, prevent health systems from becoming overwhelmed, encourage vaccination, and allow people to return to social activities in safe environments, and in that way they are a necessary tool of public health. The principle of proportionality refers to the idea that if a health pass has a potential negative impact on a person’s rights, it should be justifiable by the severity of the health risks that are being addressed. With 510 million infected people as of April 2022 and more than six million people dead (Johns Hopkins 2022), it is reasonable that some rights be restricted for the sake of public health, especially since these restrictions are temporary and they help to protect the rights of a majority of people. This point will be further explored in the remainder of the article. The principle of scientific validity evaluates whether a COVID-19 health pass is effective, timely, and accurate, which has already been analysed. And finally, to be ethical, COVID-19 passes must abide by a time-bound principle. They should be required as long as necessary to address the pandemic situation but should be withdrawn as soon as possible after the end of the pandemic. Since the end of the pandemic cannot be predicted, the use of health passes should be regularly reviewed. The decisions on whether to continue or discontinue their use should be based on these reviews.

In this section three ethical concerns will be analysed: the potential these passes have for affecting rights to privacy, equality and non-discrimination, and freedom. These are probably the main ethical concerns of those who oppose the implementation of COVID-19 health passes.

### Inequality and Discrimination

Even if a COVID-19 health pass with universal validity were to be implemented, given the unequal advancement of vaccination in the world, this would create differences between people from high-income countries, with high levels of vaccination, and those from low- and middle-income countries, with very low levels of vaccination. As of April 2022, only some 20 per cent of the population in Africa has a full vaccination schedule and in some parts of Latin America, Asia, and the Middle East less than half of the population is fully vaccinated (Holder 2022; Our World in Data 2022). While in some countries 80 per cent of the population have been vaccinated in less than a year from the beginning of vaccination, in others it is likely that reaching this level of coverage will take up to three years. In some countries, people have the option of getting vaccinated or not; in other countries, people just do not have that option because there are no vaccines. Wouldn’t all these people be discriminated against if they wanted to travel to countries with travel restrictions and that require a COVID-19 health pass, for a situation that is beyond their control? One option to avoid discrimination would be for high-income countries that are implementing COVID-19 health passes or travel restrictions to allow the entry of travellers based on the level of vaccination in their countries. Thus, for example, an exception to the restrictions that the United States has imposed is that those travellers from countries with limited vaccine availability may enter without being vaccinated but they will have to show a negative test result taken one day before departure (CDC 2022b). Another option, recommended by the WHO (2021d), is that countries do not ask for proof of COVID-19 vaccination as a condition for travel.

Beyond the inequality and discrimination that COVID-19 health passes could lead to in the context of international travel, there is a deeper concern about whether these passes might not foster a more general form of discrimination within society, since they would create, as it has been claimed, first- and second-class citizens. The vaccinated would be allowed into public places, the unvaccinated would be excluded. People who did not have a COVID-19 health pass would be, as some opponents have said, “expelled from society.”

If people do not get vaccinated it is for multiple reasons. Some people claim that their religion forbids them to be vaccinated, some others have medical conditions, such as allergies or severe anaphylactic reactions to vaccines, that make it risky to get vaccinated, many others are hesitant because they believe that given the speed with which these vaccines have been produced, all the side effects they could have are not yet known and, acting under a precautionary principle, they argue that it is better not to get vaccinated until they are fully tested (the fact that it has been said that some vaccines are in the “experimental phase” contributed to that hesitation). Others simply oppose vaccines because they have unwarranted beliefs about possible side effects or because they believe they are part of a large international conspiracy to control the lives of citizens. Whatever the reason, implementing a COVID-19 health pass would be drawing a dividing line between the vaccinated and the unvaccinated and that may lead to discrimination.

In work contexts, making work decisions about whether or not people return to work, or what work to do, based on their vaccination status is legally permitted (except when they are not vaccinated for health reasons or religious beliefs). “Vaccination status” is not a “suspect classification” on which discriminatory practices are based (such as ethnic origin, age, gender, disabilities, social status, marital status, religious beliefs, or sexual orientation, among others). However, many U.S. states are trying to change the legal standing of the vaccination status to make discrimination on that basis illegal. In Minnesota, legislators have introduced a bill that states, “it is an unfair discriminatory practice to discriminate against an individual for the individual’s vaccine status” (Minnesota Legislature 2022, section 1; see also Grossenbacher 2021). But there is a relevant difference to the traditional suspect classifications, in which there is no affectation of the rights of third parties; in the case of a person’s vaccination status there is a considerable probability that, if they are not vaccinated and get ill, they put the health and lives of their co-workers or other members of society at risk. If discrimination consists, among other things, in treating people in a different or unequal way when that treatment is arbitrary, in the sense that there are no justified moral bases for doing so, then treating people who have not been vaccinated in a different way does not constitute a form of discrimination, since there is a justified reason for such differential treatment—a reason based on relevant epidemiological evidence.

### Privacy

It is often argued that the implementation of COVID-19 health passes would constitute a violation of people’s right to privacy by allowing third parties to access information about the health status of the pass holder, which is private and confidential information. Additionally, these passes could contain sensitive medical information in some centralized database that could be vulnerable to data theft, public health surveillance, pharmacovigilance, research, or commercial misuse. The misuse of confidential medical information, in turn, could lead to discrimination based on health conditions. There are several relevant questions here, among others: 1) whether accessing information about a person’s vaccination status constitutes a violation of her right to privacy, 2) whether information on other medical conditions could be progressively included and stored in a centralized database, and 3) if the institutions or the people who handle this information give it the due protection.

One could speak of a violation of the right to privacy if the person has not consented to disclose her health information. Given that COVID-19 health passes are issued at the request of the person concerned, there is an implicit understanding that the person agrees to disclose his vaccination status each time the health pass is requested in order to enter a public place.

There is fear that the COVID-19 health pass will lead to health certificates or apps that progressively include more information about medical conditions other than COVID-19—such as HIV status, diabetes, or cardiovascular condition. However, it is likely, given the levels of mistrust and the polarization that the issuance of COVID-19 health passes has generated, that they will not be used beyond the current pandemic. Again, the implementation of digital passes should be subjected to the principles of necessity and proportionality: they should contain only the necessary information and be proportional to the governments’ proposed uses. They should not contain more information than needed, only the minimum set of data required for the purposes of the health pass. They should also follow a principle of time-boundedness: they should have legal and technical sunset clauses that allow them to be operative just as long as necessary to meet the public health needs.

This is an issue that depends on how well the protection of personal data is regulated. The contrast in how the United States and the European Union have proceeded can be significant in this regard. The Biden administration has explicitly stated that a universal central database for vaccines will not be created, due to privacy concerns (Wernau 2021). Currently, states collect that information and are required to share it with the CDC, although that information is not public (Yeginsu 2021). So, if someone is going to develop a digital vaccination certificate in the United States, she will have to obtain that information from each state; considering that many states oppose the creation of these certificates, it seems that their implementation will be very difficult. It also implies that there will not be a valid COVID-19 health pass for the entire United States and that the option of creating these digital certificates or apps will be left in the hands of private developers. Thus, IBM and CLEAR, two private companies, are developing technology for vaccine certification for use by corporations (Lee et al. 2021). In addition, there is the problem of states sharing vaccination data with different private certification platforms while maintaining the privacy of citizens. The zeal to protect privacy and the fact that there is no centralized database can heighten the risks of sharing private personal information with commercial entities.

The European Union (EU), on the other hand, has recognized possible abuses that could occur in the implementation of the Digital Green Certificate but this is based on the General Data Protection Regulation to increase public confidence in its use (Lee et al. 2021). This certificate contains necessary key information such as name, date of birth, date of issuance, relevant information about vaccine/test/recovery, and a unique identifier. This data remains on the certificate and is not stored or retained when it is verified in another EU country. All health data remains with the country that issued an EU digital COVID-19 certificate (European Commission 2022). States should be very clear from the very beginning about the proposed uses and the information required and that this information is not going to be stored in some centralized database.

Without adequate regulations and protection of personal data, there is a risk of misuse of private medical information—especially in a context in which private companies develop such passes. The problem, ultimately, lies not in the COVID passes themselves but in the legal and commercial context in which they are developed. In any case, the rights recognized in the regulations on privacy must be protected, and handling of personal data must be guaranteed.

Finally, people seem to be very concerned about the invasion of privacy issue with regard to COVID-19 health passes, yet many of these same people disclose information in very different ways without worrying too much about the private data that they provide in other contexts. Consider the personal information many people disclose in social networks such as Facebook or Instagram or when they use health and fitness apps (Véliz 2020). Of course, access to all this information must be well regulated, as in the case of digital health passes. If these data could not be well protected, the alternative would be to use a printed version with only the necessary key information. But then the risk is that these passes may be more easily forged—something already discussed.

### Autonomy, Freedom, and Public Health

Opponents of COVID-19 health passes fear that these would constitute a limitation on the freedom and personal autonomy of those who choose not to be vaccinated, either because they deliberately oppose vaccines, because it goes against their religious beliefs, for medical reasons, or simply because they have doubts about the safety of vaccines. Imposing a vaccination certificate, it is claimed, would violate the freedom of these people who have decided not to be vaccinated for whatever reason.

A first reaction to this objection would be that, in most of the countries where it has been proposed, neither vaccination nor health passes have been made mandatory—except in a few countries and generally for specific groups of people, such as public servants (Reuters 2021)—so that people remain free to decide whether or not they want to be vaccinated and whether or not they want to go to public places that require a COVID-19 health pass. However, let us assume that the freedom of those who have decided not to be vaccinated is effectively being restricted. Even so, there would be at least two conflicts to resolve: on the one hand, that of the freedom of others; on the other, a conflict between the individual freedom of those who refuse to be vaccinated and the protection of public health.

If we guarantee the right to liberty of those who refuse to be vaccinated, does this not somehow imply not guaranteeing the right to liberty of those who have been vaccinated? The introduction of a COVID-19 health pass may limit the freedom of those who refuse to be vaccinated but maximizes the freedom of all others, who have been vaccinated, by allowing them to move more freely and safely in public places. “For people who understand that widespread vaccination is our best strategy for beating the pandemic, the 25 percent of Americans who still have not received a single shot are a barrier to freedom,” says Yasmin Tayag (2021, ¶2). In this sense, COVID-19 health passes seek to guarantee the right to freedom of all those who autonomously decided to be vaccinated, that is, of the remaining 75 per cent. But something just as important, if not more, is that they seek to guarantee public health and the right to health protection.

The case of those who refuse to be vaccinated is a case of the conflict between individual freedom and public health. The COVID-19 pandemic is not the first episode of this conflict. In 1905, Henning Jacobson was fined for refusing to get vaccinated, in violation of the Massachusetts state compulsory vaccination law, which required residents to get vaccinated against smallpox. Jacobson argued that punishing him was an invasion of his freedom and that the law was “unreasonable, arbitrary, and oppressive.” One should not be compelled if it was his autonomous decision not to get vaccinated. The case reached the U.S. Supreme Court, which decided against Jacobson. The court argued that

in every well ordered society charged with the duty of conserving the safety of its members the rights of the individual in respect of his liberty may at times, under the pressure of great dangers, be subjected to such restraint, to be enforced by reasonable regulations, as the safety of the general public may demand. […] Real liberty for all could not exist under the operation of a principle which recognizes the right of each individual person to use his own [liberty], whether in respect of his person or his property, regardless of the injury that may be done to others. (*Jacobson v Massachusetts* 1905, 197 U.S. 28, ¶1)

Additionally, the court held that mandatory vaccinations are not arbitrary or oppressive as long as they do not “go so far beyond what was reasonably required for the safety of the public.” With the smallpox epidemic on the rise in the state, the vaccination requirement was “necessary in order to protect the public health and secure the public safety” (*Jacobson v Massachusetts* 1905, 197 U.S. 27, ¶1). Although the context has changed, the arguments of those who are opposed to vaccination and argue that the introduction of vaccination certificates would violate their freedoms have not changed. The answer does not need to vary much either: in the conflict between individual freedom and the protection of public health and safety, the welfare of the community weighs more heavily.

Finally, in the Jacobson case, the court also declared that

The liberty secured by the constitution of the United States to every person within its jurisdiction does not import an absolute right in each person to be, at all times and in all circumstances, wholly freed from restraint. There are manifold restraints to which every person is necessarily subject for the common good” (*Jacobson v Massachusetts* 1905, 197 U.S. 26, ¶1).

This should remind us of the many ways in which living in society restraints individual freedom. People must have a driver’s licence to operate a car, in the interest of public safety; it may be easier for people to dump their trash on the street than to put it in trashcans, but public sanitation requirements prevent such “freedom” of behaviour. Examples of how society must limit individual freedom for the common good could be multiplied. It is something of which those who oppose vaccination and health passes should be reminded.

## Conclusions

COVID-19 health passes face different practical and ethical problems. Among the practical are problems related to the accuracy of diagnostic tests and the fact that no vaccine guarantees complete immunity and that people can remain contagious even if they are vaccinated. While this is the case, a health pass would guarantee that whoever carries it has a high probability of being healthy and not being contagious. Another problem has to do with the fact that COVID-19 health passes can be forged. There are different reports that developers of these passes around the world are working to increase the security levels to protect them against possible counterfeiters. Additionally, in a universe of millions of people vaccinated in cities like New York, the fact that a few hundred people have acquired false passes constitutes a lower risk—especially a risk for those who pretend to be vaccinated but are not—compared to the benefit of guaranteeing the safety and health of the majority—who are vaccinated. The problem that health passes are not universal and are not recognized by all the states of a country or between countries can be solved as the WHO approves the different vaccines so far approved at the national level and more people in low- and middle-income countries get vaccinated. It can also be solved if states renounce “vaccine nationalism” and start accepting other countries’ vaccines. Alternatively, it could also be solved, as the WHO has advised (2021d), if countries do not require proof of COVID-19 vaccination as a condition for international travel. In sum, the practical problems do not appear to be insurmountable.

Since it has been shown that the implementation of health passes encourages vaccination, there is a powerful ethical argument in favour of this measure. It helps to reduce transmission and the risk of serious illness and death. Health passes should be implemented along with campaigns that seek to persuade people to get vaccinated. In order to do this, it is necessary for health authorities to implement campaigns to combat the infodemic that has taken place together with the COVID-19 pandemic and to seek the most persuasive methods to convince people to get vaccinated.

More challenging, in any case, are the ethical issues presented by health passes. However, it is questionable that the COVID-19 health pass constitutes a violation of the rights to privacy, non-discrimination, and freedom. With adequate regulation, based on good legislation on the protection of personal data, and following ethical principles such as necessity, proportionality, scientific validity, and time boundedness, there would be no basis to fear possible violations of these rights. The ethical justification for digital health passes will depend on the regulatory basis on which they are implemented to protect private information. There can always be printed versions of these passes that contain just the necessary key information.

The objection that health passes would be discriminatory is not justified either, because if discrimination consists of treating people in a different way when there are no good justifying moral grounds for doing so, then treating vaccinated people differently to non-vaccinated people is not discriminatory, since there is a public health justification based on epidemiological evidence for doing so. Finally, although the introduction of a health pass could be seen as a restriction of the right to freedom and the autonomy of those who have decided not to be vaccinated or not to register to obtain the health pass, there is a justification in terms of protecting the rights to freedom and safety of those who autonomously decided to get vaccinated and obtain a pass. There is also a justification in terms of protecting the right to healthcare and promoting public health. If all this is so, then there seems to be enough ethical justification for implementing COVID-19 health passes.
